# Erratum for “Feasibility of electroanatomic mapping and radiofrequency catheter ablation in Boxer dogs with symptomatic ventricular tachycardia”

**DOI:** 10.1111/jvim.16474

**Published:** 2022-08-03

**First published: April 13, 2022** https://onlinelibrary.wiley.com/doi/10.1111/jvim.16412 **Volume 36, Issue 3**

Correction to authorship information as follows.

The Journal of Veterinary Internal Medicine does not allow 2 corresponding authors or 2 first authors. Dr. Anna R. Gelzer is the corresponding author, not Dr. Cory M. Taschabrunn. Drs. Alexandra V. Crooks and Weihow Hsue contributed equally in their author roles.

